# Prognostic significance of blood immune cells in children with sepsis and establishment of a predictive model for PICU mortality: a retrospective study

**DOI:** 10.3389/fped.2024.1455216

**Published:** 2024-12-12

**Authors:** Mulan He, Qiuxia Meng, Zhixin Wei, Zhiyong Yang

**Affiliations:** Department of Pediatric Intensive Care Unit, First Affiliated Hospital of Guangxi Medical University, Guangxi, China

**Keywords:** sepsis, children, predictive model, lymphocytes, PICU mortality

## Abstract

**Objectives:**

This article aimed to investigate the correlation between blood immune cells and the prognosis in the early phase of pediatric sepsis and construct a prediction model for pediatric intensive care unit (PICU) mortality.

**Methods:**

A total of 348 children admitted with sepsis to our PICU were retrospectively collected between January 2020 and June 2024. Of these, 242 children admitted from January 2020 to October 2022 were designated as the modeling group, while 106 children admitted between November 2022 and June 2024 were designated as the prospective validation group. Peripheral blood immune-related parameters, measured from the day of PICU admission to day 7, were analyzed in the modeling group. Risk factors were identified through multivariate logistic regression and integrated into a predictive nomogram. The nomogram was then applied to the prospective validation group to assess its discrimination and calibration. The nomogram's performance was evaluated using the area under the receiver operating characteristic curves (AUC), calibration plots, and decision curve analysis for both groups.

**Results:**

Complicated with underlying diseases, invasive mechanical ventilation, increased pediatric risk of mortality score or pediatric sequential organ failure assessment score, and lymphopenia (d1) were independent risk factors for PICU mortality. The 90-day survival of patients with lymphopenia on the first day after admission was low. In addition, patients with persistent lymphopenia had higher mortality. The nomogram showed an AUC of 0.861 (95% CI: 0.813 to 0.909) in the modeling group and 0.875 (95% CI: 0.797 to 0.953) in the prospective validation group. The nomogram also performed well based on the calibration curve and decision curve analysis.

**Conclusion:**

Assessing lymphocytes within seven days of PICU admission may be conducive to identifying children with sepsis at increased mortality risk. The nomogram performed well in predicting PICU mortality among patients of interest.

## Introduction

Sepsis is defined as life-threatening organ dysfunction caused by dysregulated host systemic inflammatory and immune response to infection. In the Global Burden of Disease Study, infections have been the second leading cause of childhood deaths (1). Sepsis affects about 50 million people annually worldwide, with nearly one-half of these cases occurring in infants, children, and adolescents (2). Mortality related to pediatric severe sepsis varies greatly across geographical regions, reaching as high as 21% in North America, 29% in Europe, 32% in Australia/New Zealand, 40% in Asia, 11% in South America, and 40% in Africa (3). Most pediatric patients (67%) develop multi-organ dysfunction syndrome at the onset of sepsis, with new cases in an additional 30% of children within the initial 7 d (3). Sepsis still poses a great challenge in pediatric populations.

Immunological mechanisms play an important and complex role in the pathophysiology of sepsis. Immunologic responses evolve over the course of sepsis. The initial stages of sepsis are characterized by a proinflammatory state with activation of the innate immune system (4), releasing proinflammatory cytokines, such as IL-1, IL-2, IL-6, IFN-γ, and TNF-α (5). Immune cell activation and inflammatory responses can cause immune-mediated organ damage. When this phase is not treated promptly, overactivation of the immune system can occur, accompanied by an excessive anti-inflammatory and potentially immunosuppressive response, potentially increasing the risk of secondary infections (6). Immunosuppression is related to poor outcomes in pediatric sepsis, yet its relationship with the corresponding prognosis remains a research gap.

To date, the international academic community has revised the definition and diagnosis of sepsis three times. The third definition (Sepsis-3) was unanimously passed by the 45th Society of Critical Care Medicine in 2016 (7). In this version, emphasis is placed on the mechanism, the identification of disease severity, and the timely intervention in clinical treatment. In addition to mechanistic studies, assessing illness severity and mortality risk for patients with sepsis becomes a routine in critical care medicine. Many risk assessment systems for critically ill children are widely employed in clinical practice (8–10), such as Pediatric Risk of Mortality Score Ⅲ (PRISM Ⅲ), Pediatric Critical Illness Score (PCIS), Oxford Acute Severity of Illness Score (OASIS), and the most commonly used Sequential Organ Failure Assessment (SOFA) score, serving as the diagnostic criteria of sepsis 3.0 (11, 12). Despite its popularity, the SOFA score has been reported to have shortcomings (e.g., low sensitivity) (13, 14). In 2013, Johnson et al. (15) constructed the OASIS, which has a good predictive effect on the prognosis of critically ill patients in ICUs (16). Due to the presence of ten non-specific indicators and lack of laboratory data, its ability to predict the fatality risk is limited. A reliable prognosis assessment tool for sepsis is urgently warranted.

This study retrospectively analyzed the clinical feature data of children with sepsis in PICU. We aimed to clarify the correlation between the dynamic changes in blood immune cells within the first 7 days of admission to the PICU and the prognosis of pediatric sepsis. Subsequently, the risk factors associated with PICU mortality in children were further evaluated. Finally, we constructed a prediction model and created a visual nomogram to guide clinical decision-making.

## Methods

### Study setting and population

Data were collected from a consecutive cohort of 348 children with sepsis admitted to the Department of Pediatric Intensive Care Unit in the First Affiliated Hospital of Guangxi Medical University (Guangxi, China) between January 2020 and June 2024. A total of 242 children, enrolled between January 2020 and October 2022, were included as the modeling group, while 106 children enrolled between November 2022 and June 2024 were included as the prospective validation group. Specifically, participants included patients aged one month to 18 years diagnosed with sepsis according to the International Pediatric Sepsis Consensus Conference criteria published in 2005 (17). Patients with substantial missing data related to medical records or a PICU stay <24 h were excluded. In total, 242 patients were enrolled in the study.

### Data collection

The following information was collected: (1) Epidemiological data, including age, sex, height, weight, and body mass index (BMI). (2) Laboratory data such as lactate, procalcitonin (PCT), C-reactive protein (CRP), Interleukin-6 (IL-6), D-dimer, lymphocyte subsets, and routine blood tests. (3) Comorbidities (e.g., congenital heart disease, solid or hematologic malignancies, post-liver transplantation, post-hematopoietic stem cell transplantation, genetic metabolic diseases, epilepsy, rheumatic immune system diseases, congenital immune deficiency, and biliary atresia). (4) Organ function support during PICU stay, including mechanical ventilation therapy and continuous renal replacement therapy (CRRT). (5) Other relevant data involving admission time, discharge time, PICU admission duration, PICU discharge time, diagnosis, infection site, and total hospitalization costs. PCIS, PRISM, and pediatric SOFA (pSOFA) were used to assess the severity of illness among patients on PICU admission.

### Outcome

The primary outcomes included PICU mortality, 90-day mortality, duration of PICU stay, and total length of hospital stay. Notably, the children who signed the waiver of treatment and were discharged automatically were also considered as PICU hospitalized deaths.

### Definitions

According to the new 2016 adult definitions and criteria (Sepsis-3), sepsis and septic shock in adults are defined as follows:

Sepsis: life-threatening organ dysfunction resulting from a dysregulated host response to infection (7).
Septic shock: a subset of sepsis characterized by particularly profound circulatory, cellular and metabolic abnormalities associated with a greater risk of mortality than sepsis alone (7).

The definition of pediatric sepsis remains an immense challenge and is debated. The last published definitions for sepsis, severe sepsis, and septic shock in children are based on the 2005 International Pediatric Sepsis Consensus Conference (17).

Pediatric sepsis: Systemic inflammatory response syndrome (SIRS) in the presence or as the result of a suspected or proven infection.
Diagnostic criteria for SIRS: Meeting at least two of the following criteria indicates SIRS, and either abnormal temperature or leukocyte count is required:
  (1) Pyrexia (> 38.5°C) or hypothermia (< 36°C);
  (2) Age-dependent tachycardia or bradycardia;
  (3) Tachypnea or need for mechanical ventilation;
  (4) Abnormal white blood cell count or >10% immature neutrophils.
Infection: Suspected or proven (by positive culture, tissue sample, or polymerase chain reaction test) infection resulting from a pathogen or clinical syndrome associated with a high probability of infection. Evidence of infections includes positive findings on clinical examination, imaging studies, or laboratory tests, such as white blood cells in normally sterile body fluid, chest x-ray consistent with pneumonia, purpuric or petechial rash, and purpura fulminant.
Pediatric severe sepsis: Sepsis and cardiovascular dysfunction, respiratory dysfunction, or two or more non-cardiorespiratory organ system dysfunctions.
Pediatric septic shock: Sepsis in the presence of cardiovascular dysfunction. Septic shock is defined as one of the manifestations such as hypotension, receipt of vasoactive medication, and impaired perfusion despite fluid resuscitation.

### Grouping situation

The children in the modeling group were further divided into survival and non-survival groups according to mortality outcomes during PICU hospitalization. According to the lymphocyte count on d1, these pediatric patients were divided into the lymphopenia group (lymphocyte count < 1.5 × 10^9^/L) and the lymphocyte normal group (lymphocyte count ≥ 1.5 × 10^9^/L). The lymphopenia group was further categorized into two subgroups: the temporary lymphopenia group (lymphocyte count returned to normal on d3 or d7) and the persistent lymphopenia group (lymphocyte count still decreased on d7).

### Statistical analysis

All statistical analyses were performed using R 4.0.1 (R Core Team, Vienna, Austria) and SPSS 26.0 (IBM, Chicago, IL, USA). Additionally, they were set as two-sided tests with a 0.05 significance level. Kolmogorov-Smirnov test was used to test the normality of continuous variables. Data for continuous variables with normal distribution were presented as mean ± standard deviation (*x* ± *s*), and the differences between groups were assessed for significance using the student-*t* test. In contrast, data with skewed distribution were expressed as median (25% percentile, 75% percentile) [M: (25%P, 75%P)], and the significance of differences was evaluated using the Mann–Whitney test. Data for categorical variables were presented as frequencies (percentages) [*n* (%)], and the *χ*^2^ test was employed to assess significant differences. The multivariate logistic regression model involving variables with *P* < 0.100 was developed, and stepwise selection was applied using the likelihood ratio test and Akaikes information criterion as the stopping rule. The regression results were considered to define a nomogram model responsible for predicting PICU mortality using the RMS package. The calibration curves combined with the Hosmer-Lemeshow test were introduced to evaluate the accuracy. Specifically, the Receiver Operating Characteristic (ROC) curve was used to verify the discrimination, and the decision curve analysis (DCA) served to evaluate the net benefit for the clinical practicability of the nomogram model. The Kaplan-Meier method was deployed to analyze the survival between lymphopenia and lymphocyte normal groups within 90 d, and the Log Rank test was conducted to explore the difference in survival curves between the two groups.

## Results

### The clinical characteristics and laboratory findings of participants in the modeling group

The general clinical characteristics of the 242 patients with sepsis in the modeling group were shown in Table 1. The median age was 4.05 (0.98, 9.34) years. Specifically, 145 were boys (59.9%). The median BMI was 15.11 (13.60, 17.16) kg/m^2^. In addition, 124 cases (51.2%) had underlying diseases. The most prevalent underlying diseases were malignant tumor or hematological diseases (55 cases), post-hematopoietic stem cell transplantation (21 cases), congenital heart disease (10 cases), post-liver transplantation (10 cases), and rheumatic immune system diseases (8 cases). The primary infection sites included the lung (186 cases, 76.9%), nervous system (32 cases, 13.2%), and abdomen (16 cases, 6.6%). A total of 154 (63.6%) patients required invasive mechanical ventilation, with a median duration of 40.25 h (0.00, 145.25). Thirteen (5.4%) patients were treated with CRRT. The median length of hospital stay was 20 d (10.00, 32.00), and the median duration of PICU stay was 6 d (3.00, 11.25). Notably, 107 (44.2%) patients died within 90 d after sepsis diagnosis.

**Table 1:** Clinical characteristics and laboratory findings of 242 children with sepsis in the modeling group.

Characteristics [*n* (%) or median (IQR)]	Total (*n* = 242)	Survival group (*n* = 150)	Non-survival group (*n* = 92)	*P* value
Demographics				
Age [median (IQR)]	4.05 (0.98–9.34)	3.10 (0.80–8.70)	5.90 (1.15–11.38)	0.052
Male (%)	145 (59.9)	94 (62.7)	51 (55.4)	0.265
BMI [median (IQR)]	15.11 (13.60–17.16)	15.28 (13.74–17.30)	14.86 (13.84–16.91)	0.666
Rural population (%)	167 (69.0)	103 (68.7)	64 (69.6)	0.883
Underlying diseases (%)				0.019
Malignant tumor or hematological diseases	55 (22.7)	32 (21.3)	23 (25)	
Post-hematopoietic stem cell transplantation	21 (8.7)	7 (4.7)	14 (15.2)	
Congenital heart disease	10 (4.1)	6 (4.0)	4 (4.3)	
Post-liver transplantation	10 (4.1)	3 (2.0)	7 (7.6)	
Rheumatic immune system diseases	8 (3.3)	5 (3.3)	3 (3.3)	
Epilepsy	7 (2.9)	4 (2.7)	3 (3.3)	
Genetic metabolic diseases	6 (2.5)	4 (2.7)	2 (2.2)	
Congenital immunodeficiency	5 (2.1)	2 (1.30)	3 (3.3)	
Biliary atresia	2 (0.8)	2 (1.3)	0 (0.0)	
Infection site (%)				0.509
Lung	186 (76.9)	116 (77.3)	70 (76.1)	
Nervous system	32 (13.2)	17 (11.3)	15 (16.3)	
Abdomen	16 (6.6)	12 (8.0)	4 (4.3)	
Urinary system	7 (2.9)	4 (2.7)	3 (3.3)	
Body surface	1 (0.4)	1 (0.7)	0 (0.0)	
Severity of illness [median (IQR)]				
PCIS score	86.00 (80.00–92.00)	90.00 (80.00–94.00)	80.00 (80.00–91.50)	<0.001
PRISM score	5.00 (2.00–9.00)	4.00 (2.00–8.00)	8.00 (5.00–12.00)	0.001
pSOFA score	4.00 (2.00–6.00)	3.00 (2.00–5.00)	5.00 (3.25–10.00)	<0.001
Laboratory examination [median (IQR)]				
Lactate (mmol/L)	2.30 (1.54–3.72)	2.10 (1.48–3.62)	3.00 (1.86–5.23)	0.001
PCT (ng/ml)	1.82 (0.34–7.21)	1.93 (0.42–10.04)	1.27 (0.28–5.89)	0.354
CRP (mg/L)	29.67 (6.99–88.07)	37.82 (6.80–84.52)	28.26 (8.22–99.46)	0.995
IL-6 (pg/ml)	287.30 (41.27–553.00)	129.30 (28.10–553.00)	505.00 (98.01–553.00)	0.008
D-dimer (ng/ml)	3,559.00 (732.50–4,271.00)	2,644.50 (648.50–4,271.00)	4,271.00 (1,701.00–4,271.00)	0.079
Clinical outcome				
Total hospital stay [median (IQR)]	20.00 (10.00–32.00)	21.00 (13.00–34.00)	10.50 (6.00–27.00)	<0.001
Duration of PICU stay [median (IQR)]	6.00 (3.00–11.25)	6.00 (3.00–12.00)	6.00 (2.00–10.75)	0.585
In-hospital mortality (%)	92 (38.0)	0 (0.0)	92 (100.0)	
28-day mortality (%)	89 (36.8)	2 (1.3)	87 (94.6)	<0.001
90-day mortality (%)	107 (44.2)	16 (10.7)	91 (98.9)	<0.001
Others				
Mechanical ventilation (%)	154 (63.6)	70 (46.7)	84 (91.3)	<0.001
Duration of mechanical ventilation [median (IQR)]	40.25 (0.00–145.25)	0.00 (0.00–95.00)	82.50 (32.44–191.25)	<0.001
CRRT (%)	13 (5.4)	4 (2.7)	9 (9.8)	0.035
Hospitalization costs				
Total hospital expenses [median (IQR)]	63,190.07 (35,577.30–1,30,793.08)	68,434.69 (38,741.46–1,11,928.37)	58,805.11 (30,426.91–1,71,686.21)	0.761

IQR, interquartile range; BMI, body mass index; PCIS, pediatric critical illness score; PRISM**,** pediatric risk of mortality; pSOFA, pediatric sequential organ failure assessment; PCT, procalcitonin; CRP, C-reactive protein; IL-6, interleukin-6; PICU, pediatric intensive care unit; CRRT, continuous renal replacement therapy.

Compared to the survival group, the number and duration of patients receiving mechanical ventilation are significantly greater (*P* = 0.008, <0.001). More non-survivors received CRRT (*P* < 0.001). Furthermore, the non-survival group exhibited significantly lower PCIS score (*P* < 0.001, higher PRISM score (*P* = 0.001), and greater pSOFA score (*P* < 0.001) than the survival counterpart, as well as significantly increased levels of serum IL-6 (*P* = 0.008).

### Comparison of immune-related parameters in routine blood test of 242 children with sepsis

**Figure 1:**
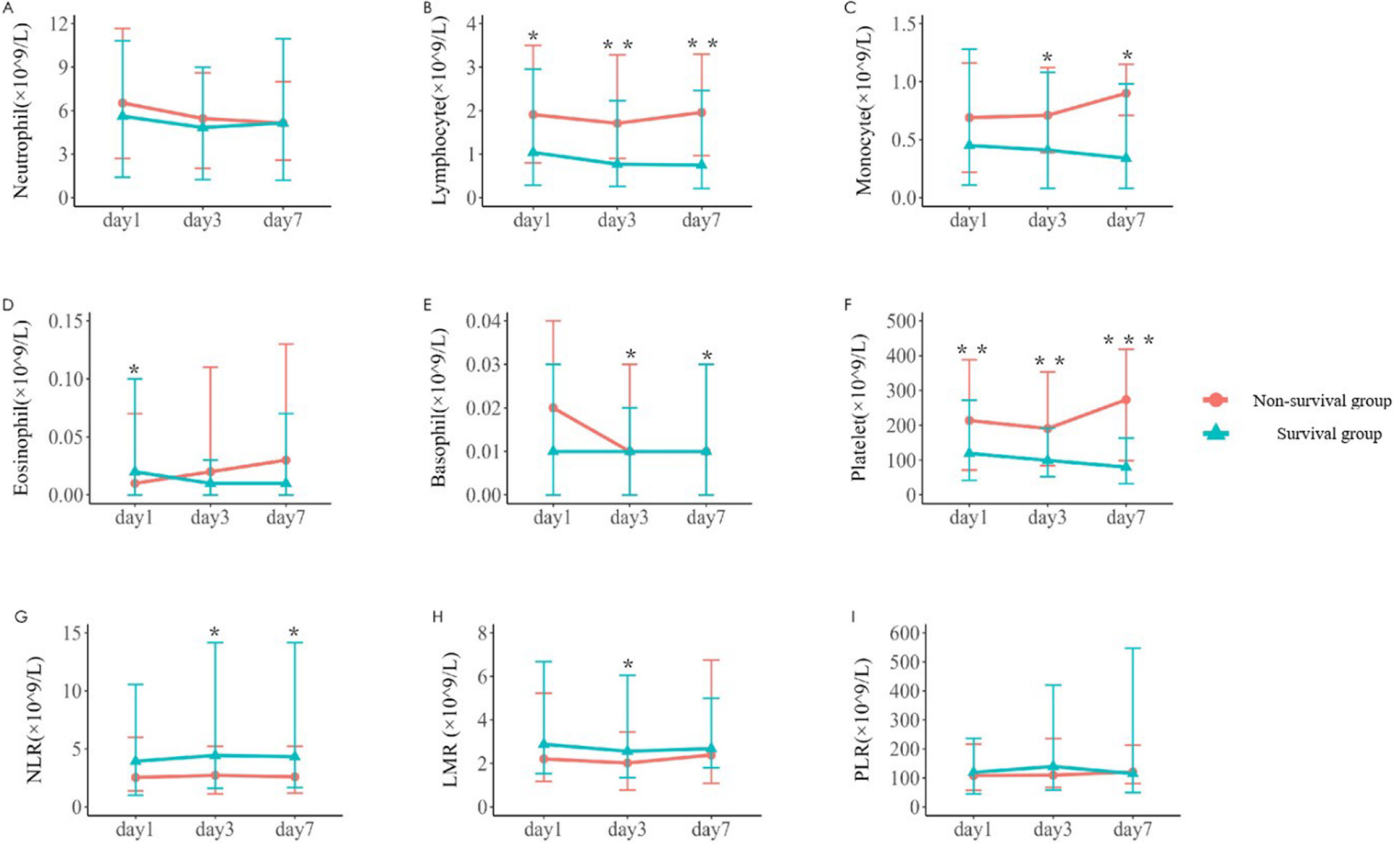
Variations in parameters related to blood routine immune cells in the two groups [**(A–F)** The number of neutrophil, lymphocyte, monocyte, eosinophil, basophil, and platelet on d1, d3, and d7 after admission in the survival group and the non-survival group. **(G–I)** The neutrophil-to-lymphocyte ratio (NLR), lymphocyte-to-monocyte ratio (LMR) and platelet-to-lymphocyte ratio (PLR) on d1, d3, and d7. *P < 0.05, **P < 0.01, ***P < 0.001.]

The changes of immune cells in the blood routine of 242 children with sepsis in the modeling group were analyzed on d1, d3, and d7 after admission. Compared with survivors, non-survivors underwent significant decreases in the lymphocyte counts on d1, d3, and d7, the number of monocytes on d3 and d7, the number of eosinophils on d7, the basophil counts on d3 and d7, and the platelet counts on d1, d3, and d7, as shown in Table 2 and Figure 1. In addition, significant increases were observed in the neutrophil-to-lymphocyte ratio (NLR) on d3 and d7 and lymphocyte to monocyte ratio (LMR) on d3. The neutrophil count and platelet-to-lymphocyte ratio (PLR) displayed no significant differences between the two groups on d1, d3, and d7 (*P* > 0.05) (Table 2).

**Table 2:** Comparison of immune-related parameters in 242 children with sepsis in the modeling group.

Absolute value of immune cells [median (IQR)]	Total	Survival group	Non-survival group	*P* value
Neutrophil (×10^9^/L)				
d1 (*n* = 242)	6.35 (1.95–11.23)	6.53 (2.70–11.67)	5.62 (1.41–10.82)	0.353
d3 (*n* = 234)	5.15 (1.81–8.79)	5.46 (2.03–8.61)	4.84 (1.24–8.98)	0.571
d7 (*n* = 218)	5.15 (2.09–8.52)	5.15 (2.58–8.00)	5.15 (1.20–10.96)	0.805
Lymphocyte (×10^9^/L)				
d1 (*n* = 242)	1.63 (0.67–3.39)	1.91 (0.80–3.50)	1.04 (0.2–2.95)	0.006
d3 (*n* = 234)	1.43 (0.33–2.80)	1.71 (0.90–3.28)	0.77 (0.26–2.23)	<0.001
d7 (*n* = 218)	1.53 (0.58–3.13)	1.96 (0.97–3.30)	0.75 (0.21–2.46)	<0.001
Monocyte (×10^9^/L)				
d1 (*n* = 242)	0.61 (0.17–1.18)	0.69 (0.22–1.16)	0.45 (0.11–1.28)	0.157
d3 (*n* = 234)	0.59 (0.20–1.12)	0.71 (0.39–1.12)	0.41 (0.08–1.08)	0.023
d7 (*n* = 218)	0.66 (0.21–1.11)	0.71 (0.90–1.15)	0.34 (0.08–0.98)	0.002
Eosinophil (×10^9^/L)				
d1 (*n* = 242)	0.01 (0.00–0.07)	0.01 (0.00–0.07)	0.02 (0.00–0.10)	0.671
d3 (*n* = 234)	0.01 (0.00–0.08)	0.02 (0.00–0.11)	0.01 (0.00–0.03)	0.076
d7 (*n* = 218)	0.02 (0.00–0.11)	0.03 (0.00–0.13)	0.01 (0.00–0.07)	0.011
Basophil (×10^9^/L)				
d1 (*n* = 242)	0.02 (0.00–0.03)	0.02 (0.00–0.04)	0.01 (0.00–0.03)	0.396
d3 (*n* = 234)	0.01 (0.00–0.03)	0.01 (0.00–0.03)	0.01 (0.00–0.02)	0.015
d7 (*n* = 218)	0.01 (0.00–0.03)	0.01 (0.00–0.03)	0.01 (0.00–0.03)	0.017
Platelet (×10^9^/L)				
d1 (*n* = 242)	149.60 (59.3–336.45)	213.85 (71.60–388.38)	119.35 (42.20–272.03)	0.001
d3 (*n* = 234)	142.85 (60.8–290.58)	190.75 (83.78–353.08)	99.15 (52.23–191.73)	<0.001
d7 (*n* = 218)	173.90 (66.1–358.40)	273.30 (98.60–418.20)	80.00 (32.15–162.70)	<0.001
NLR				
d1 (*n* = 242)	2.96 (1.23–6.90)	2.54 (1.38–6.01)	3.94 (1.00–10.56)	0.154
d3 (*n* = 234)	2.96 (1.23–6.90)	2.73 (1.13–5.22)	4.4 (1.61–14.15)	0.018
d7 (*n* = 218)	3.11 (1.38–6.67)	2.60 (1.19–5.22)	4.33 (1.67–14.15)	0.002
LMR				
d1 (*n* = 242)	2.76 (1.33–6.38)	2.21 (1.18–5.23)	2.88 (1.54–6.67)	0.072
d3 (*n* = 234)	2.32 (1.17–5.39)	2.02 (0.78–3.44)	2.56 (1.35–6.05)	0.020
d7 (*n* = 218)	2.62 (1.50–5.11)	2.39 (1.09–6.75)	2.68 (1.80–4.99)	0.232
PLR				
d1 (*n* = 242)	112.32 (55.50–222.17)	108.28 (57.82–216.18)	119.41 (45.27–236.21)	0.117
d3 (*n* = 234)	120.21 (63.85–257.27)	109.59 (67.27–235.84)	139.65 (58.52–419.53)	0.111
d7 (*n* = 218)	120.83 (70.58–245.76)	121.17 (81.01–213.07)	115.29 (49.46–547.49)	0.242

IQR, interquartile range; NLR, neutrophil-to-lymphocyte ratio; LMR, lymphocyte-to-monocyte ratio; PLR, platelet-to-lymphocyte ratio.

### Analysis of lymphocyte subsets in 165 children with sepsis within 7 d of PICU admission

One hundred and sixty-five children with sepsis in the modeling group were involved in the peripheral blood lymphocyte subsets analysis within 7 d after admission to PICU. Compared with the survival group, the median absolute counts of total T cells, CD4^+^ T cells, and CD8^+^ T cells in the non-survival group were significantly decreased (*P* < 0.05), as listed in Table 3.

**Table 3:** Analysis of lymphocyte subsets within 7 days of admission to PICU in 165 children with sepsis of the modeling group.

Lymphocyte subsets (/μl) [median (IQR)]	Total	Survival group (*n* = 101)	Non-survival group (*n* = 64)	*P* value
Total T cell count	1,062.00 (213.50–1,968.00)	1,398.00 (657.50–2,325.00)	265.00 (83.09–1,081.50)	<0.001
CD4^+^T cell count	477.00 (95.50–1,046.50)	659.00 (262.50–1,393.00)	115.5 (34.25–548.75)	<0.001
CD8^+^T cell count	477.00 (103.50–793.50)	585.00 (256.50–947.50)	166.50 (38.50–526.50)	<0.001
CD4^+^Tcell/CD8^+^Tcell	1.17 (0.73–1.92)	1.30 (0.82–2.05)	0.98 (0.62–1.47)	0.019

IQR, interquartile range.

### Univariate and multivariate logistic regression analyses for evaluating factors influencing PICU mortality

The clinical outcomes (death or alive) of 242 children with sepsis hospitalized in PICU were used as binary variables. Subsequently, age, gender, BMI, rural population, complicated with underlying disease, infection site, invasive mechanical ventilation, CRRT, PCIS, PRISM, pSOFA scores on d1, and auxiliary examination [Lactate, PCT, CRP, IL-6, D-dimer, lymphopenia (<1.5 × 10^9^/L), thrombocytopenia (<100 × 10^9^/L), etc.] on d1 were included in the univariate and multivariate logistic regression analyses. Univariate logistic regression analysis revealed that increased age [OR: 1.066 (1.011, 1.124)], the presence of underlying disease [OR: 2.338 (1.370, 3.990)], receipt of invasive mechanical ventilation [OR: 12.00 (5.430, 26.520)], CRRT [OR: 3.958 (1.182, 13.249)], dropped PCIS [OR: 0.936 (0.900, 0.973)], escalated PRISM [OR: 1.186 (1.112, 1.265)], mounted pSOFA score [OR: 1.186 (1.112, 1.265)], elevated lactate [OR: 1.186 (1.112, 1.265)], as well as the presence of lymphopenia [OR: 2.467 (1.449, 4.201)] and thrombocytopenia [OR: 1.762 (1.030, *P* < 0.05]. 3.013)] on d1 were the risk factors for the pediatric mortality. According to the results of multivariate logistic regression analysis, having underlying diseases [OR: 2.696 (1.258, 5.775)], invasive mechanical ventilation [OR: 11.992 (4.613, 31.171)], increased PRISM [OR: 1.110 (1.013, 1.216)], growing pSOFA score [OR: 1.154 (1.001, 1.216)], and lymphopenia [OR: 2.747 (1.299, 5.809)] (d1) constituted independent risk factors for death in children with sepsis admitted to PICU (Table 4).

**Table 4:** Univariate and multivariate logistic regression analyses of prognostic factors in 242 children with sepsis.

Influence factors	Univariate analysis				Multivariate analysis			
	*B*	Wald	OR (95% CI)	*P* value	*B*	Wald	Corrected OR (95%CI)	*P* value
Age	0.064	5.603	1.066 (1.011–1.124)	0.018				
Male	−0.300	1.239	0.741 (0.437–1.256)	0.266				
BMI	−0.015	0.374	0.985 (0.939–1.034)	0.541				
Rural population	0.042	0.022	1.043 (0.594–1.830)	0.883				
Complicated with underlying disease	0.849	9.695	2.338 (1.370–3.990)	0.002	0.992	6.510	2.696 (1.258–5.775)	0.011
Mechanical ventilation	2.485	37.722	12.00 (5.430–26.520)	<0.001	2.484	25.979	11.992 (4.613–31.171)	<0.001
CRRT	1.376	4.980	3.958 (1.182–13.249)	0.026				
PCIS	−0.067	11.250	0.936 (0.900–0.973)	0.001				
PRISM	0.171	27.073	1.186 (1.112–1.265)	<0.001	0.104	4.979	1.110 (1.013–1.216)	0.026
pSOFA score	0.272	32.552	1.313 (1.196–1.441)	<0.001	0.143	3.914	1.154 (1.001–1.216)	0.048
Lactate	0.193	11.329	1.212 (1.084–1.356)	0.001				
PCT	−0.006	1.102	0.994 (0.984–1.005)	0.294				
CRP	0.000	0.004	1.000 (0.996–1.004)	0.949				
IL-6	0.000	2.601	1.000 (1.000–1.001)	0.107				
D-dimer	0.000	0.247	1.000 (1.000–1.000)	0.619				
d1 lymphopenia	0.903	11.061	2.467 (1.449–4.201)	0.001	1.010	6.993	2.747 (1.299–5.809)	0.008
d1 thrombocytopenia	0.566	4.277	1.762 (1.030–3.013)	0.039				

BMI, body mass index; PCIS, pediatric critical illness score; PRISM**,** pediatric risk of mortality; pSOFA, pediatric sequential organ failure assessment; PCT, procalcitonin; CRP, C-reactive protein; IL-6, interleukin-6; CRRT, continuous renal replacement therapy.

### The forest map of independent risk factors for PICU mortality in children with sepsis

**Figure 2:**
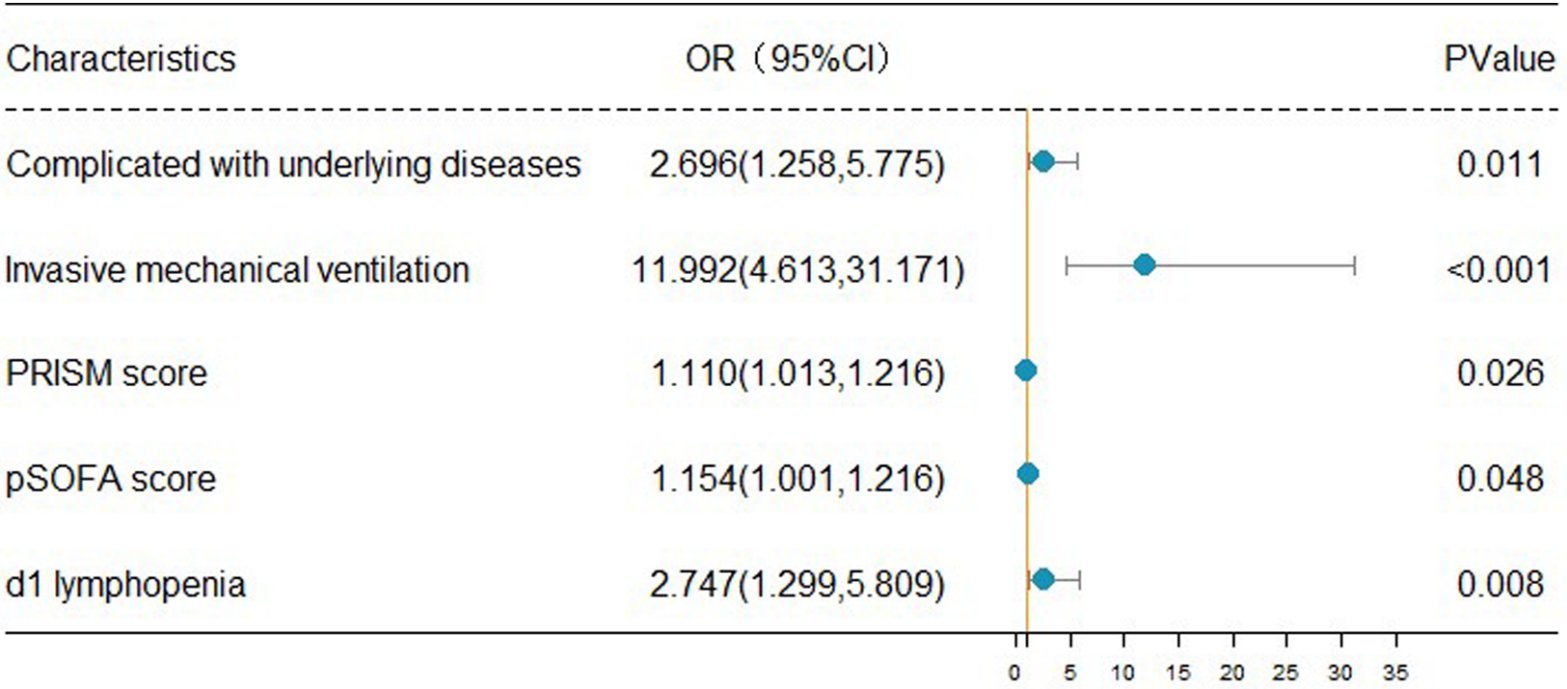
Forest map of independent risk factors for PICU mortality in children with sepsis.

For a clear and intuitive representation of the results, the five independent prognostic factors, including underlying diseases, invasive mechanical ventilation, increased PRISM, increased pSOFA score, and lymphopenia (d1), were screened out from multivariate logistic regression analysis and plotted as a forest map (Figure 2).

### Comparison of clinically relevant parameters between the lymphocytopenia group and the non-lymphocytopenia group in 242 children with sepsis

The patients in the modeling group were further divided into lymphopenia group (lymphocyte count <1.5 × 10^9^/L) and lymphocyte normal group (lymphocyte count ≥1.5 × 10^9^/L) on the first day after admission. By comparing the relevant clinical data between the two groups, we found that the children in the lymphopenia group were older than those in the lymphocyte normal group. The PRISM and 90-day mortality rates were higher in the lymphopenia group than in the lymphocyte normal group (Table 5).

**Table 5:** Comparison of clinically relevant parameters between the lymphocytopenia group and the non-lymphocytopenia group (242 cases).

Characteristics [*n* (%) or median (IQR)]	D1 lymphopenia group (<1.5 × 10^9^/L) (*n* = 114)	D1 lymphocyte normal group (≧1.5 × 0^9^/L) (*n* = 128)	*Z/χ* ^2^	*P* value
Age [median (IQR)]	6.95 (2.88, 11.03)	1.90 (0.50, 6.98)	−5.046	<0.001
Male (%)	71 (62.3)	74 (57.8)	0.501	0.479
Total hospitalization days [median (IQR)]	21.00 (10.00, 34.25)	17.50 (10.00, 30.00)	−1.148	0.251
Stay in PICU [median (IQR)] (Day)	6.00 (3.00, 13.25)	6.00 (3.00, 10.75)	−1.497	0.134
Invasive mechanical ventilation (%)	72 (63.2)	82 (64.1)	0.021	0.884
Duration of Ventilator [median (IQR)]	45.75 (0.00, 188.25)	39.50 (0.00, 91.73)	−1.372	0.170
CRRT (%)	9 (7.9)	4 (3.1)	2.699	0.100
PRISM [median (IQR)]	8.00 (4.00, 2.00)	4.00 (0.00, 8.00)	−4.514	<0.001
PCIS [median (IQR)]	90.00 (80.00, 92.00)	82.00 (80.00, 92.00)	−1.288	0.198
pSOFA score [median (IQR)]	4.00 (3.00, 7.00)	4.00 (2.00, 6.00)	−1.363	0.173
PICU mortality (%)	56 (49.1)	36 (28.1)	11.282	0.001
90-day mortality (%)	61 (53.5)	46 (35.9)	7.548	0.006

IQR, interquartile range; PCIS, pediatric critical illness score; PRISM**,** pediatric risk of mortality score; pSOFA, pediatric sequential organ failure assessment; PICU, pediatric intensive care unit; CRRT, continuous renal replacement therapy.

**Figure 3:**
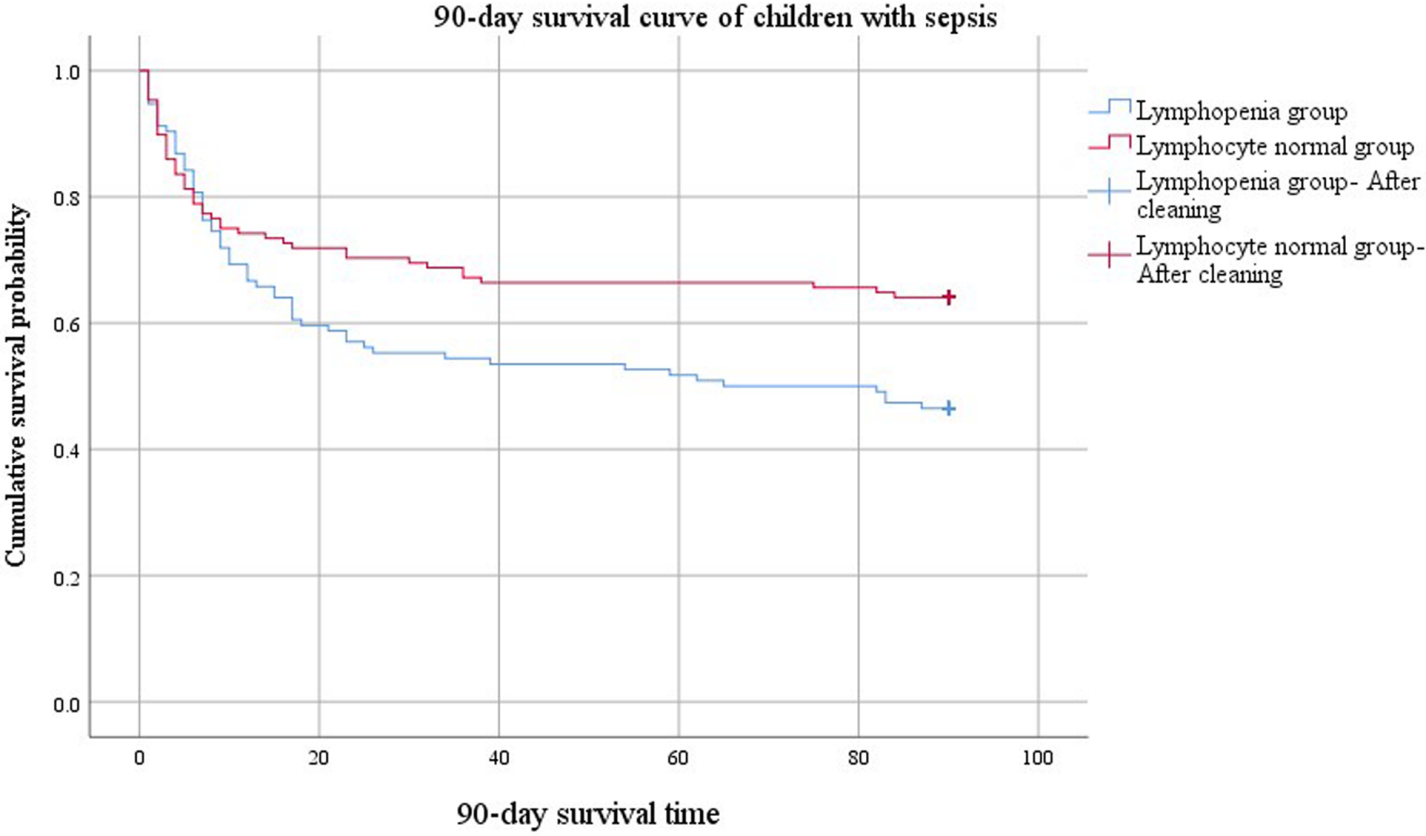
90-day survival curves of the lymphopenia group and non-lymphopenia group.

The comparison of 90-day survival curves between the two groups demonstrated worsened outcomes in the lymphopenia group (Figure 3). When the cumulative survival probability of the lymphopenia group was 50%, the survival duration was 65 d. In contrast, the cumulative survival probability in the lymphocyte normal group exceeded 50%, with a minimum value of 64%, and a prolonged survival duration (84 d) was observed, indicating a better prognosis for this group. The difference in 90-day survival curves between lymphopenia and lymphocyte normal groups compared using the Log Rank (Mantel-Cox) test was statistically significant (*χ*^2^ = 5.849, *P* = 0.016).

### Effect of persistent lymphopenia on the prognosis of children with sepsis

A total of 111 children with sepsis from the lymphopenia group who had complete data on lymphocyte counts on d1, d3, and d7 after admission were separated into two subgroups: temporary lymphopenia group (lymphocyte counts returned to normal on d3 or d7, *n* = 48) and persistent lymphopenia group (lymphocyte counts remained <1.5 × 10^9^/L on d7, *n* = 63). By comparing the clinical characteristics between the subgroups, significant differences were found regarding age, length of hospital stay, PRISM, and PICU mortality. Children in the persistent lymphopenia group were older than those in the temporary lymphopenia group. In addition, they had a longer total length of hospital stay, higher mortality risk score, and greater PICU mortality (Table 6).

**Table 6:** Comparison of clinically relevant indicators between the temporary and persistent lymphopenia groups.

Characteristics [*n* (%) or median (IQR)]	Persistent lymphopenia group (*n* = 63)	Temporary lymphopenia group (*n* = 48)	*t/Z/χ^2^*	*P* value
Age [median (IQR)]	8.27 ± 4.48	5.13 ± 4.04	3.683	<0.001
Male (%)	40 (63.5)	29 (60.4)	0.110	0.741
Total hospitalization length [median (IQR)] (Day)	26.00 (13.00, 47.00)	18.50 (9.00, 28.00)	−2.067	0.039
Stay in PICU [median (IQR)] (Day)	6.00 (4.00, 13.00)	7.00 (2.25, 71.00)	−0.110	0.912
Invasive mechanical ventilation (%)	41 (65.1)	30 (37.5)	0.079	0.779
Duration of Ventilator [median (IQR)]	40.00 (0.00, 168.00)	61.00 (0.00, 206.60)	−0.277	0.781
CRRT (%)	6 (9.5)	3 (6.3)	0.076	0.783
PRISM [median (IQR)]	9.00 (6.00, 12.00)	5.50 (2.00, 8.00)	−3.863	<0.001
PCIS [median (IQR)]	90.00 (80.00, 92.00)	90.00 (80.00, 96.00)	−0.556	0.579
pSOFA score [median (IQR)]	4.50 (3.00, 7.25)	4.00 (2.00, 6.75)	−1.084	0.278
PICU mortality (%)	37 (58.7)	18 (37.5)	4.912	0.027
90-day mortality (%)	37 (58.7)	22 (45.8)	1.820	0.177

IQR, interquartile range; BMI, body mass index; PCIS, pediatric critical illness score; PRISM, pediatric risk of mortality score; pSOFA, pediatric sequential organ failure assessment; PICU, pediatric intensive care unit; CRRT, continuous renal replacement therapy.

### Nomogram construction of PICU mortality prediction model for children with sepsis

**Figure 4:**
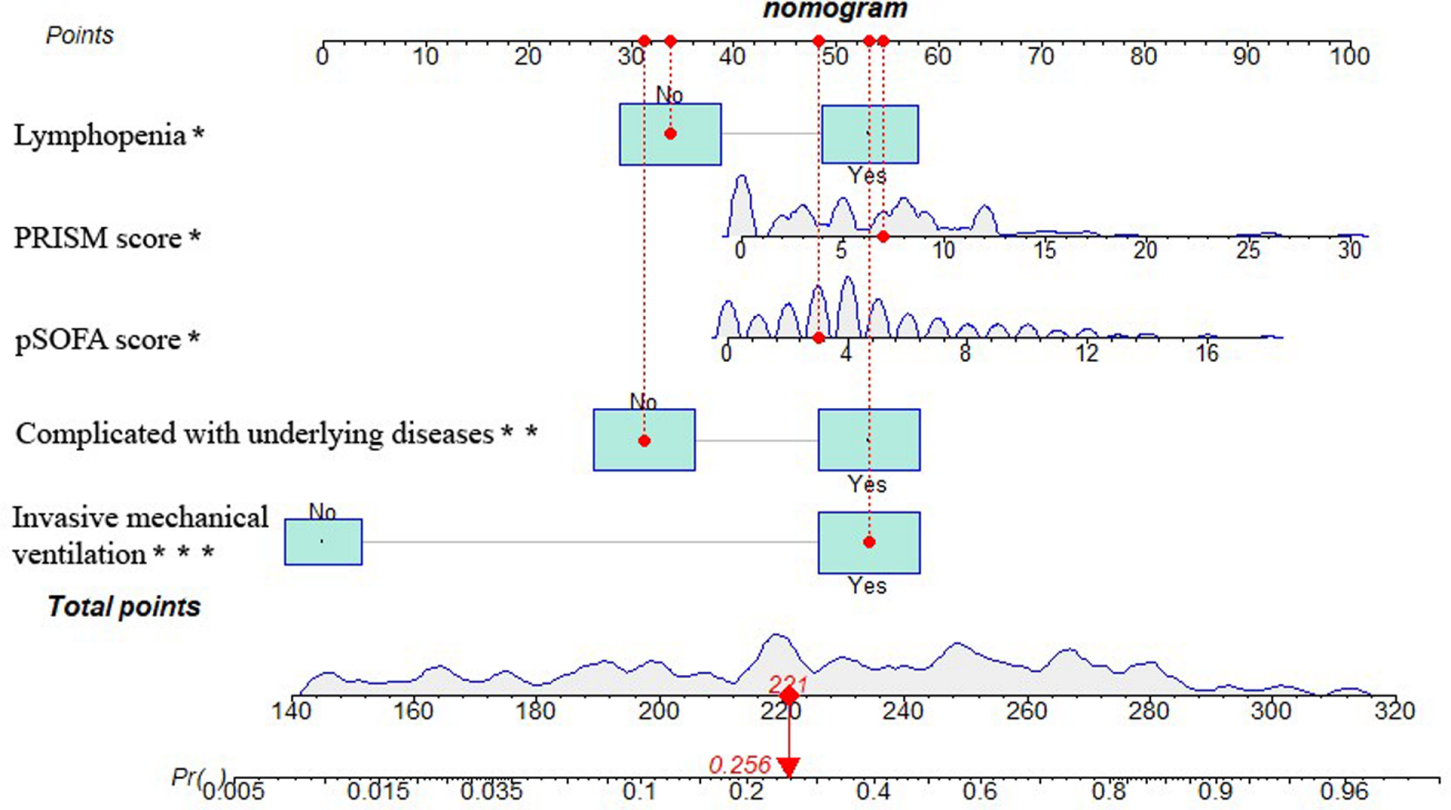
Nomogram of PICU mortality prediction model for children with sepsis. [Example illustrating the use of the model was marked in red: Taking a 1.90-year-old child with sepsis as an example, the pSOFA score is 3 (corresponding points = 48), no lymphopenia occurs (corresponding points = 34), PRISM is 7 (corresponding points = 55), invasive mechanical ventilation is required (corresponding points = 53), and no underlying diseases is present (corresponding points = 31), totaling 221 points, which corresponds to a mortality rate of about 25.6%.].

The five independent predictors (lymphopenia, PRISM, pSOFA score, complicated with underlying diseases, and invasive mechanical ventilation) selected by multivariate logistic regression analysis were further integrated into a visualized nomogram (Figure 4). Each predictor index corresponded to a value and score on the scale, and the total score was obtained by summing up the scores of all indicators. The total score was the PICU mortality projection of children with sepsis at the bottom. The clinician can give an individualized evaluation of the risk of PICU death for children undergoing sepsis according to the total points.

### Comparison of baseline clinical characteristics between the modeling group and prospective validation group

To assess the performance of the model, a cohort was collected as the prospective validation group. A comparison of baseline characteristics between the modeling group (*n* = 242) and prospective validation group (*n* = 106) was summarized in Table 7. No signiﬁcant difference was observed between the two groups across any of the clinical indicators (*P >* 0.05).

**Table 7:** Comparison of baseline clinical characteristics between the modeling group and prospective validation group.

Characteristics [*n* (%) or median (IQR)]	Modeling group (*n* = 242)	Prospective validation group (*n* = 106)	*Z/χ* ^2^	*P* value
Age [median (IQR)]	4.05 (0.98, 9.34)	5.88 (1.92, 9.67)	−1.392	0.164
Male (%)	145 (59.9)	64 (30.6)	0.007	0.936
Complicated with underlying disease	118 (48.8)	58 (54.7)	1.046	0.306
D1 lymphocyte count (×10^9^/L)	1.63 (0.67, 3.39)	1.55 (0.46, 2.36)	−1.491	0.136
Total hospitalization days [median (IQR)]	20.00 (10.00, 32.00)	19.50 (11.75, 28.50)	−0.003	0.997
Stay in PICU [median (IQR)] (Day)	6.00 (3.00, 11.25)	6.00 (3.00, 11.00)	−0.295	0.768
Invasive mechanical ventilation (%)	88 (36.4)	29 (27.4)	2.678	0.102
Duration of Ventilator [median (IQR)]	40.25 (0.00, 145.25)	54.06 (0.00,177.38)	−1.052	0.293
PRISM [median (IQR)]	5.00 (2.00, 9.00)	7.00 (3.00, 11.00)	−1.796	0.073
pSOFA score [median (IQR)]	4.00 (2.00, 6.00)	4.00 (3.00, 6.00)	−1.464	0.143
PICU mortality (%)	150 (62.0)	73 (68.9)	1.518	0.218

### Prospective validation of the predictive model

**Figure 5:**
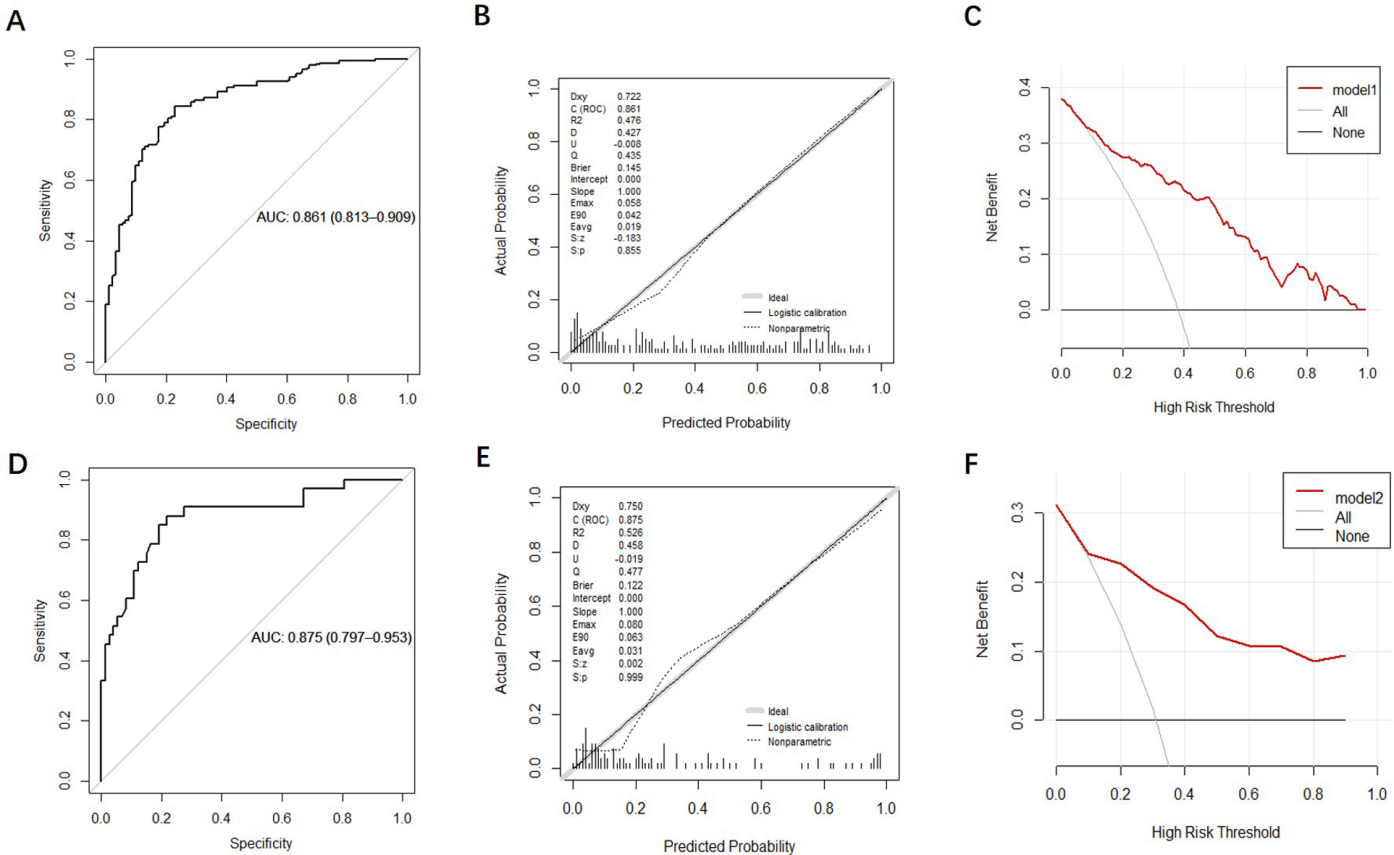
Validation and evaluation of the nomogram model. [**(A–C)**: ROC curve, calibration chart and decision curve analysis of the modeling group; **(D–F)**: ROC curve, calibration curve, and decision curve analysis for the prospective validation group.].

The discrimination of the nomogram was evaluated using the ROC curve. The nomogram based on data in the modeling group gave an AUC of 0.861 (95% CI: 0.813–0.909) for predicting mortality (Figure 5A). Similarly, it gave an AUC of 0.875 (95% CI: 0.797–0.953) in the prospective validation group (Figure 5D). Calibration curves were plotted to evaluate the accuracy of the nomogram. For both groups, the nomogram showed good agreement with observed mortality based on calibration curves (Figures 5B,E). The Hosmer-Lemeshow test gave a *P* = 0.855 in the modeling group while it gave a *P* = 0.2809 in the prospective validation group. Despite the slight deviation, the results indicated high consistency in the predicted probability and observed probability. DCA showed promising clinical utility for the nomogram, based on the modeling group (Figure 5C) and prospective validation group (Figure 5F). Specifically, when the threshold probability exceeded 10%, the net clinical benefit predicted using the nomogram was more pronounced in actively treated patients.

## Discussion

Sepsis is a leading contributor to global mortality and has been declared a global health priority by the World Health Organization (18). Pediatric sepsis mortality varies reportedly from 4% to 50% depending on the severity of illness, risk factors, and geographical locations (19). Among 242 children with sepsis in the current research, the in-hospital fatality rate was 38.0%, consistent with the previous report. A 2019 systematic review revealed a downward trend in case-fatality rates for children with severe sepsis and septic shock (20). To date, the early recognition and screening tools for clinical deterioration of pediatric sepsis remain challenging.

Historically, a biphasic pattern has been reported in the sepsis death distribution; specifically, an initial early peak appears several days after sepsis recognition due to inadequate fluid resuscitation, resulting in cardiac and pulmonary failure, followed by a late peak arising from persistent organ injury or failure at several weeks (21). Conventionally, therapeutic strategies for sepsis have focused on inhibiting the early hyperinflammatory phase. However, due to the concurrence of an immune suppression state and persistent inflammation, persistent, recurrent, secondary, and nosocomial infections develop, leading to poorer outcomes and increased long-term mortality (22). Sepsis-induced immune suppression impacts both cellular effectors of the innate and adaptive immune systems.

In terms of innate immunity, neutrophils can act as antigen-presenting cells to synchronize the innate and adaptive immune response during infection (23). Mature neutrophils undergo apoptosis throughout the sepsis progression (24). Our study found almost normal absolute neutrophil counts at d1, d3, and d7 and no significant differences between the survival and non-survival groups. These inconsistent results suggested the necessity of investigating the effect of neutrophils on the prognosis of pediatric sepsis. Monocyte populations undergo various changes during sepsis. Under pathological conditions, monocytes possess a decreased capacity to release inflammatory mediators (such as TNF, IL-1, IL-6, and IL-12). In comparison, their ability to produce inhibitory cytokines (IL-10, etc.) is unimpaired and enhanced in some cases (25). In this study, the monocyte count in the non-survival group was lower than that in the survival group on d1, d3, and d7, especially on d3 and d7. This finding indicated the suppressed proinflammatory function and depletion of monocytes in the course of sepsis, aggravating the immunosuppression. The wider role of platelets as “first responders” in host defense and their important functions in innate and adaptive immunity have been recognized (26, 27). Thrombocytopenia is prevalent in patients with sepsis during PICU and neonatal intensive care unit stay and is associated with poor prognosis (27, 28). The results of this study unveiled that platelet counts were significantly decreased in non-survivors, and the presence of thrombocytopenia increased the mortality risk in young patients with sepsis. These results indicated that most innate immune cells were suppressed in the early stage of sepsis in children, potentially affecting the prognosis.

In addition to diminished innate function, adaptive immunity was also impaired. Lymphocyte populations are involved in mounting a successful adaptive immune response in all aspects of infection. Sepsis initiates as a cytokine storm and quickly progresses to a state of lymphopenia (29, 30). The important role of lymphocyte apoptosis contributing to immunosuppression in sepsis pathogenesis has been recognized (31). Splenocytes harvested from deceased patients with sepsis demonstrate reduced numbers of CD4^+^ and CD8^+^ lymphocytes that emanate from substantial apoptosis (32). Among 165 children with sepsis who completed lymphocyte subset analysis within 7 d of PICU admission in our study, the median absolute values of total T cells, CD4^+^ T cells, and CD8^+^ T cells in the non-survival group were significantly lower than those in the survival group. Compared with the survivors, the non-survivors had significant decreases in lymphocyte counts on d1, d3, and d7. Children with lymphocyte count <1.5 × 10^9^/L suffered relatively high mortality. Considering the influence of underlying diseases, multivariate logistic regression analysis revealed that lymphopenia (d1) was an independent risk factor for death in children with sepsis. The mortality rate was relatively high in patients with lymphopenia despite the absence of underlying diseases. These results indicated that the depletion of adaptive immune cells also occurred in the early phase of pediatric sepsis, affecting the prognosis. Multiple studies have demonstrated that adaptive and innate immune suppression in children within the first 2 d of septic shock was associated with adverse outcomes (33, 34). We should consider the adverse consequences of continued lymphopenia. The occurrence of lymphopenia 4 d after the onset of sepsis was linked with the development of secondary infection and served as a predictive factor for long-term mortality one year after the initial septic episode (35). In the current paper, children in the persistent lymphopenia group had a prolonged total length of hospital stay and relatively high PICU mortality. For PICU clinicians, regardless of the underlying disease status, dynamic monitoring of lymphocyte count is promising as a simple and feasible indicator of immunosuppression to predict the prognosis of sepsis, guide doctor-patient communication, and adjust clinical treatment strategies.

The commonly used prognostic screening tools in clinical practice for pediatric sepsis have distinct advantages and disadvantages. SOFA score is the fundamental clinical criteria to identify individuals meeting Sepsis-3; despite this developed criteria, specific criteria for children are lacking (36). Schlapbach et al. (37) developed a pSOFA by adjusting the cardiovascular and renal components of SOFA score for age in 2016. Notably, the SOFA score is based on adult studies, resulting in a research gap of recognized standards suitable for children, especially for cardiovascular and renal function. The main body of these studies is single-center surveys with small sample size. Peer verification is still needed before the promotion and use of the pSOFA. Pollack et al. (38) developed and validated a third-generation pediatric physiology-based score for mortality risk, namely the PRISM III. This score has 17 physiologic variables subdivided into 26 ranges in 1996. PRISM III is built upon several improvements over the original PRISM. Clinical studies have confirmed its promising potential in predicting disease severity and mortality risk (39, 40). PCIS was formulated according to the national conditions of China in 1995 by the Emergency Subspecialty Group of the Pediatric Society of the Chinese Medical Association and the Subspecialty Group of Pediatrics of the Chinese Society of Emergency Medicine. This score has advantages such as ease of operation and good data accessibility, even in secondary medical institutions. Consequently, it is widely used in PICUs at all levels in China. Despite its capacity to indicate the severity of illness, PCIS cannot accurately predict the prognosis of disease in children (8). Our study found that among 242 children with sepsis in PICUs, non-survivors had higher severity of illness, lower PCIS, higher PRISM, and higher pSOFA score than survivors. Multivariate Logistic regression analysis demonstrated that increased PRISM and pSOFA score were independent risk factors for in-hospital death in children admitted to PICUs. The results of this paper further verified the important clinical value of pSOFA and PRISM-III in evaluating the prognosis of sepsis in children.

However, the pathological mechanism of sepsis is complex and highly heterogeneous. The predictive value of a single indicator for sepsis mortality is limited. In this study, an integrated predictive model (i.e., nomogram) was constructed based on logistic regression analysis to reliably predict mortality. Nomogram is an intuitive chart allowing for directly predicting prognosis in patients. This visual prediction model included five independent risk factors for mortality (such as lymphopenia, PRISM, pSOFA, complicated with underlying diseases, and invasive ventilation) screened by multivariate logistic regression analysis. Our nomogram showed AUC values greater than 0.8 in both the modeling and prospective validation cohorts, indicating strong discrimination and predictive accuracy. Additionally, the calibration curve further confirmed a good fit. The DCA also indicated that the nomogram had promising potential for clinical application with great net clinical benefit. In sum, this nomogram may guide clinicians in allocating resources appropriately to improve patient outcomes. Zeng et al. (41) developed a nomogram that may reliably predict 90-day mortality in adult patients with sepsis and obtained an AUC above 0.8 based on age, international normalized ratio, lactate, and thrombomodulin. Combined with our study, the nomogram model for predicting mortality allows for a more comprehensive evaluation of the severity and prognosis of pediatric sepsis, and the required detection indicators are clinically readily available data, favoring its application and promotion.

This study has limitations. First of all, the results of this retrospective study can only suggest the correlation between the changes in blood immune parameters and the clinical prognosis of sepsis, yet they cannot confirm a clear causal relationship. Second, this investigation is a single-center study from a provincial third-class A general hospital. As a result, information bias may exist in the source of children with sepsis, lacking representativeness at a national level. Third, the number of patients included in the study was relatively small, potentially introducing bias in the statistical analysis results. Finally, the developed prediction model of mortality risk in children with sepsis needs further improvement using a multicenter study with external validation.

## Data Availability

The raw data supporting the conclusions of this article will be made available by the authors, without undue reservation.
